# Orchid Biochemistry 2.0

**DOI:** 10.3390/ijms23126823

**Published:** 2022-06-19

**Authors:** Jen-Tsung Chen

**Affiliations:** Department of Life Sciences, National University of Kaohsiung, Kaohsiung 811, Taiwan; jentsung@nuk.edu.tw

In the Special Issue entitled “Orchid Biochemistry”, researchers explored the biochemistry and molecular mechanisms of pigment formation, flower scent, bioactive compounds, plant–microbial interaction, as well as aspects of biotechnology, and these studies have greatly enriched the understanding in the field of orchid biology [1]. In the second volume of this Special Issue, entitled “Orchid Biochemistry 2.0”, one literature review and nine original research articles were published, and the Special Issue provides further insight into several critical subtopics, including reproduction biology, functional genomics in secondary metabolites, as well as polysaccharides and orchid mycorrhizae.

## 1. Pollination and Flowering Biology

Orchids are ideal models for the study of pollination biology due to their diverse flowers that adapted structurally and chemically during evolution. Brzosko et al. studied plant–pollinator interactions using *Neottia ovata* (L.) Bluff & Fingerh., a generalist orchid, as a model to explore the impact of soil parameters on flower structure and nectar chemistry [2]. The authors found that carbon and the ratio of carbon to nitrogen might be the most important factor affecting flower structure and nectar composition. Furthermore, Brzosko et al. investigated the impact of the flower structure and nectar chemistry in *Epipactis palustris* (L.) Crantz, another generalist orchid that is pollinated by over 100 species of pollinators, on reproductive success [3]. The authors concluded that there are significant differences in nectar chemical properties between natural and anthropogenic populations of *E. palustris* and pointed out that future study is needed to clarify the most critical factor between pollinator differentiation and soil characteristics.

The molecular basis of flowering in orchids is yet to be fully understood, and one of the principle questions is the function of MADS-box genes. Lucibelli et al. conducted an in silico differential expression analysis to identify two YABBY *DL*/*CRC* homologs in *Phalaenopsis equestris* (Schauer) Rchb.f., namely *PeDL1* and *PeDL2* [4]. It was found that *PeDL2* regulates the differentiation of labella, and this finding enriches our knowledge of the regulatory network for orchid flower development. *Apetala 2* (*AP2*) is a gene that codes for a transcription factor, and it belongs the *AP2*/*EREBP* gene family, which plays key roles in regulating growth and development in plants. Zeng et al. identified 14 homologs of *AP2* in a popular medicinal orchid, *Dendrobium officinale* Kimura & Migo, namely *DoAP2-1* to *DoAP2-14* [5]. After subcellular localization and functional analysis of these genes, it was found that *DoAP2* may encode transcriptional repressors and be involved in flower development, stress response and other biological activities.

## 2. Functional Genomics in Secondary Metabolites and Polysaccharides

Terpenes, the largest family of plant secondary metabolites, possess a range of vital roles in plant growth and development. Yu et al. studied the gene family of terpene synthase (TPS) in *D. officinale*. The authors identified 34 TPS genes (*TPS*s) and analyzed their expression patterns, and it was found that the predominantly expressed organ is flowers. Among these genes, *DoTPS10* was selected for further investigation under abiotic stress, and it was found that the targeted subcellular localization of DoTPS10 is in chloroplasts, and in in vitro test, it was shown to convert geranyl pyrophosphate into linalool specifically [6]. Huang et al. contributed a review article for the comprehensive analysis of the evolution pathway of *TPS*s in orchids [7]. The authors refined the phylogeny of *TPS*s and suggested that the driving force of evolution in each sub-*TPS* gene family might be different and chiefly depend on pollinator attraction, stress tolerance, and/or genotype-specific characteristics. Among terpenes, geraniol is commercially important and is involved in plant–pollinator interaction and stress biology in nature. Zhao et al. identified *DoGES1*, a gene which encodes geraniol synthase, using genomic annotation data of *D. officinale* [8]. The authors studied the subcellular localization and functions of DoGES1 and finally concluded that *DoGES1* was highly expressed in the petals of semi-open flowers and effectively controlled geraniol biosynthesis in *D. officinale*. The MYB (myeloblastosis) family of transcription factors can be found in animals and plants. Among them, MYB2 acts as a transcriptional repressor in anthocyanin biosynthesis. Lim et al. studied the role of MYB2 in *Dendrobium bigibbum* Lindl., and it was demonstrated that the accumulation of purple color in leaves is associated with the increased expression of *MYB2* [9].

Polysaccharides are critical constituents in medicinal orchids, and theoretically, the chemical modification of acetylation or deacetylation could certainly affect their bioactivities. Si et al. investigated the homologs of *REDUCED WALL ACETYLATION* (*RWA*), which encode acetyltransferases, in *D. officinale*, and three *DoRWA* were identified. Eventually, DoRWA3 was demonstrated to be involved in transferring acetyl groups to polysaccharides [10].

## 3. Orchid–Fungus Symbiosis

Orchid mycorrhizae are symbiotic relationships between orchids and fungi, particularly in the early stage of seed germination and the subsequent development of seedlings. Chen et al. investigated the effect of an exogenous gibberellic acid (GA_3_) on the symbiotic germination of *D. officinale* in vitro [11]. The results indicated that exogenous GA_3_ had a dose-dependent effect on the establishment of the symbiotic relationship, and it was shown that it might act on the complicated signaling networks or biosynthetic pathways of hormones.

## 4. Conclusions and Perspectives

Altogether, this present Special Issue makes certain progress in revealing the secrets of orchid biology, and interestingly, five out of ten articles chose to conduct their research on a medicinal orchid, *Dendrobium officinale*. It is worth mentioning that the field of functional genomics for the exploration of biosynthesis and signaling networks in secondary metabolites is also currently receiving attention. Additionally, in this Special Issue, our knowledge regarding pollination biology, flowering mechanisms and symbiosis in orchids was expanded; however, there are still numerous questions that have yet to be answered. Shortly, the picture of orchid biology will be more complete due to the efforts of orchid researchers in the application of advanced and high-throughput technologies such as genome editing and integrative multi-omics.
